# Functional coherence metrics in protein families

**DOI:** 10.1186/s13326-016-0076-y

**Published:** 2016-06-23

**Authors:** Hugo P. Bastos, Lisete Sousa, Luka A. Clarke, Francisco M. Couto

**Affiliations:** LaSIGE, Faculdade de Ciências, Universidade de Lisboa, Lisboa, Portugal; CEAUL, Departamento de Estatística e Investigação Operacional, Faculdade de Ciências, Universidade de Lisboa, Lisboa, 1749-016 Portugal; BioISI - Biosystems & Integrative Sciences Institute, Faculdade de Ciências, Universidade de Lisboa, Lisboa, 1749-016 Portugal

## Abstract

**Background:**

Biological sequences, such as proteins, have been provided with annotations that assign functional information. These functional annotations are associations of proteins (or other biological sequences) with descriptors characterizing their biological roles. However, not all proteins are fully (or even at all) annotated. This annotation incompleteness limits our ability to make sound assertions about the functional coherence within sets of proteins. Annotation incompleteness is a problematic issue when measuring semantic functional similarity of biological sequences since they can only capture a limited amount of all the semantic aspects the sequences may encompass.

**Methods:**

Instead of relying uniquely on single (reductive) metrics, this work proposes a comprehensive approach for assessing functional coherence within protein sets. The approach entails using visualization and term enrichment techniques anchored in specific domain knowledge, such as a protein family. For that purpose we evaluate two novel functional coherence metrics, mUI and mGIC that combine aspects of semantic similarity measures and term enrichment.

**Results:**

These metrics were used to effectively capture and measure the local similarity cores within protein sets. Hence, these metrics coupled with visualization tools allow an improved grasp on three important functional annotation aspects: completeness, agreement and coherence.

**Conclusions:**

Measuring the functional similarity between proteins based on their annotations is a non trivial task. Several metrics exist but due both to characteristics intrinsic to the nature of graphs and extrinsic natures related to the process of annotation each measure can only capture certain functional annotation aspects of proteins. Hence, when trying to measure the functional coherence of a set of proteins a single metric is too reductive. Therefore, it is valuable to be aware of how each employed similarity metric works and what similarity aspects it can best capture. Here we test the behaviour and resilience of some similarity metrics.

**Electronic supplementary material:**

The online version of this article (doi:10.1186/s13326-016-0076-y) contains supplementary material, which is available to authorized users.

## Background

Over the last two decades functional annotation systems have been providing annotations for numerous proteins as well as other gene products. One of the most common steps used in functional annotation is the use of sequence alignment algorithms to compare sequences and find homologies from which functions can be extrapolated. Usually, lists of proteins (or other gene products) result from the output of many high-throughput technologies. Therefore, not only is it important to identify common functions in those sets of proteins but also to quantify how functionally related the proteins are in order to increase understanding of the involvement of biological systems [1, 2].

The Gene Ontology (GO) project aims to provide generically consistent descriptions for the molecular phenomena in which gene products are involved [3]. For over a decade the increasing popularity and consequent growth of GO has led to its adoption and prevalent use in annotation projects. Consequently, this pervasiveness has enabled and motivated the development of several semantic similarity metrics [4–6]. Semantic similarity can be defined as the quantity that reflects the closeness in meaning of two concepts in an ontology. However, the semantic similarity between two proteins, which can be annotated with several GO terms is commonly called “functional similarity” since it is the functional annotation terms that are being compared. More recently, several metrics focusing specifically on measuring the functional cohesiveness of a set of proteins (or gene products) through their annotations have been developed. These metrics for the assessment of functional coherence using annotations are commonly based on the previously developed groupwise semantic similarity approaches.

One of those metrics, GS^2^ [7], uses a set-based approach and was developed with computational efficiency in mind to measure gene set functional similarity based on GO terms. The GS^2^ algorithm ranks annotation terms using a simple gene counting method and then compares each gene with the remaining genes with respect to the distribution of functional annotations. This simple measure can only capture similarity trends within gene sets and can not precisely assess similarity. Despite that, GS^2^ has performed well when compared with the semantic similarity pairwise measure of [8].

On the other hand, another set of three different metrics: *average seed degree*, *total length* and *relative seed degree* were developed by [9], for the assessment of functional coherence in gene sets based on the topological properties of GO-derived graphs. The procedure leading to these metrics relies on building GO subgraphs that subsume each gene set annotation (for each GO aspect), whereas each node is a GO term and each edge is an *is_a* relationship between terms. Subsequently, those graphs are further enriched by adding genes, as a new type of node, associated to the original GO nodes, and additional new edges are created between GO terms whenever these share gene annotations. The original term-to-term edges are weighted using the Information Content [10] difference between both terms while the new edges created after addition of the gene nodes to the graph are statistically weighted based on the total number of edges in the graph and the number of supporting genes for each particular edge. Hence, this approach handles the issue at hand both from an annotation enrichment perspective and an annotation relationship perspective. Steiner trees are then extracted from the graphs and the sum of all edge lengths is minimized for all possible subgraphs. The aforementioned three metrics are then applied to these trees. The *average seed degree* averages, for a full tree, the counts of the number of genes associated to the seed terms thus reflecting a global measure of enrichment. On the other hand the *total length* metric reflects the overall relatedness of functions by performing the sum of the length of all edges in a tree. The *relative seed degree* metric combines the aspects described above as a ratio. The methodology performs well, but like other GO-evaluation methodologies, its metrics are dependent on the gene annotation state.

The GO-based functional dissimilarity (GFD) metric [11] approaches the problem of functional coherence in gene sets by considering that each gene can encode several proteins with different functions. In this metric, for each gene set, only the most common and specific function is chosen as being the most globally cohesive function. In this approach, genes are represented as sets to which a simple counting edge-based measure ratio is applied and that aims at equating both gene relatedness and specificity. The actual GFD is then the minimum of dissimilarity possible for all representations of a given set of genes. Like the previous metrics this one also depends on the completeness of the annotations used in order to provide accurate measurements. Furthermore, by considering only the most common and specific function in a gene set the authors are effectively discarding potential non-related functions that would cause noise, however at the cost of disregarding multi-functional associations in gene sets.

Furthermore, and despite not being exactly a system for measuring functional coherence in gene sets, RuleGO [12] provides a service that statistically compares and characterizes two disjointed gene sets. Underneath it runs a rule-based system that incrementally iterates the list of GO terms annotating the two input gene sets and verifies at each step if a new co-occurrence rule can be created. Much like the typical gene enrichment systems, this system also performs over-representation tests on the rules created and only rules corresponding to a p-value below a given statistical significance threshold (after multiple testing correction) are considered. This process results in multi-attribute rules containing annotation terms and respective support indexes and evaluation parameters that can be used in the characterization of the disjointed gene sets. In this methodology rules are evaluated by *length* (number of genes in a rule premise) representing support, by *depth* (normalized sum of the GO graph levels where terms in the rule appear) representing specificity and by an additional quality measure.

A different approach is taken by [13] where functional coherence in gene sets is assessed with the help of the biological literature. Here, term-by-gene matrices are constructed with entries derived from weighted frequencies of the terms across a collection of abstracts (biological literature). The genes are then represented as vectors and the similarity between them is calculated as the cosine of the vector angles. Thus, a pair of genes would have a cosine score of 1.0 if they shared the exact same abstracts in the collection. Gene sets in this method were deemed functionally coherent when cosine values above a given threshold (0.6) were often found with significances measured by a statistical test (Fisher’s exact test). This threshold was chosen based on the distribution of similarity cosine scores in 1,000 random gene sets. Hence, functional coherence here is derived essentially from the supporting literature, thus making the method sensitive to the quality of the document corpus used. Regardless, the method was used to obtain results similar to those produced by another literature-based functional coherence assessing method [14].

Since functional annotation quality is paramount, [15] developed a system to provide an annotation confidence score for genome annotations. The system operates on the basis of a genome comparison approach whereby annotations in a target genome are scored in comparison with a reference genome. The gene alignments across genomes are made via the BLAST tool with adjustments for expected number of genes (different organisms have different gene counts) and phylogenetic distance (closer genomes typically share more genes than distant ones). However, actual annotation similarity is derived from free-text annotations which are converted into word vectors that enable the calculation of a simple cosine similarity measure. Both sequence similarity and annotation similarity are combined into a single metric by applying statistical techniques.

Despite the existence of these types of metrics the protein annotation landscape is often very heterogeneous in terms of quality, specificity and completeness. Annotation quality is related with the annotation method and source used, e.g. defined by the different GO evidence codes associated to each annotation. Annotation specificity relates to how specific or general an annotation term is, and when in a protein set there is a clear disproportion between general term annotations and specific annotations, that set can be said to suffer from annotation incompleteness.

In this work we concern ourselves mostly with the aspects of annotation completeness and specificity. Given that functional similarity is derived from semantic similarity approaches over the annotation terms, it is also relevant to define the concept of *annotation agreement* as a measure of annotation homogeneity for a given set of proteins. This metric, will naively measure the coherence of a given set based on the fraction of shared annotation terms between all proteins in the set, and thus will be highly susceptible to the lack of annotation completeness. We use this measure as a baseline whereas we introduce other metrics to characterize the state of known functional similarity of a given set and gauge the potential state of annotation incompleteness. Hence, in this work *functional coherence* is defined as a measure of functional closeness (similarity) among all proteins in a set given the current functional annotations within that protein set.

## Methods

A functional annotation is defined as a pairing between a gene product (protein) identifier and a term providing some functional description. In this study, only the *molecular function* term annotations from GO were considered because the aim of this work lies closer to studying one-dimensional annotation (as proposed by [16]) at the molecular functional level in enzymes. Ideally, the functional annotations over a given protein set should allow us to infer biological relationships within the set. In order to achieve that, it is convenient to have metrics that enable us to compare how similar (or dissimilar) annotations are within a given protein set. However, considering the GO DAG structure it becomes apparent that measuring functional relatedness via annotation is not a trivial matter. Therefore, in order to help make such assertions regarding functional relatedness, three main annotation aspects were considered: *completeness*, *agreement* and *coherence*.

### Completeness

**Fig. 1:**
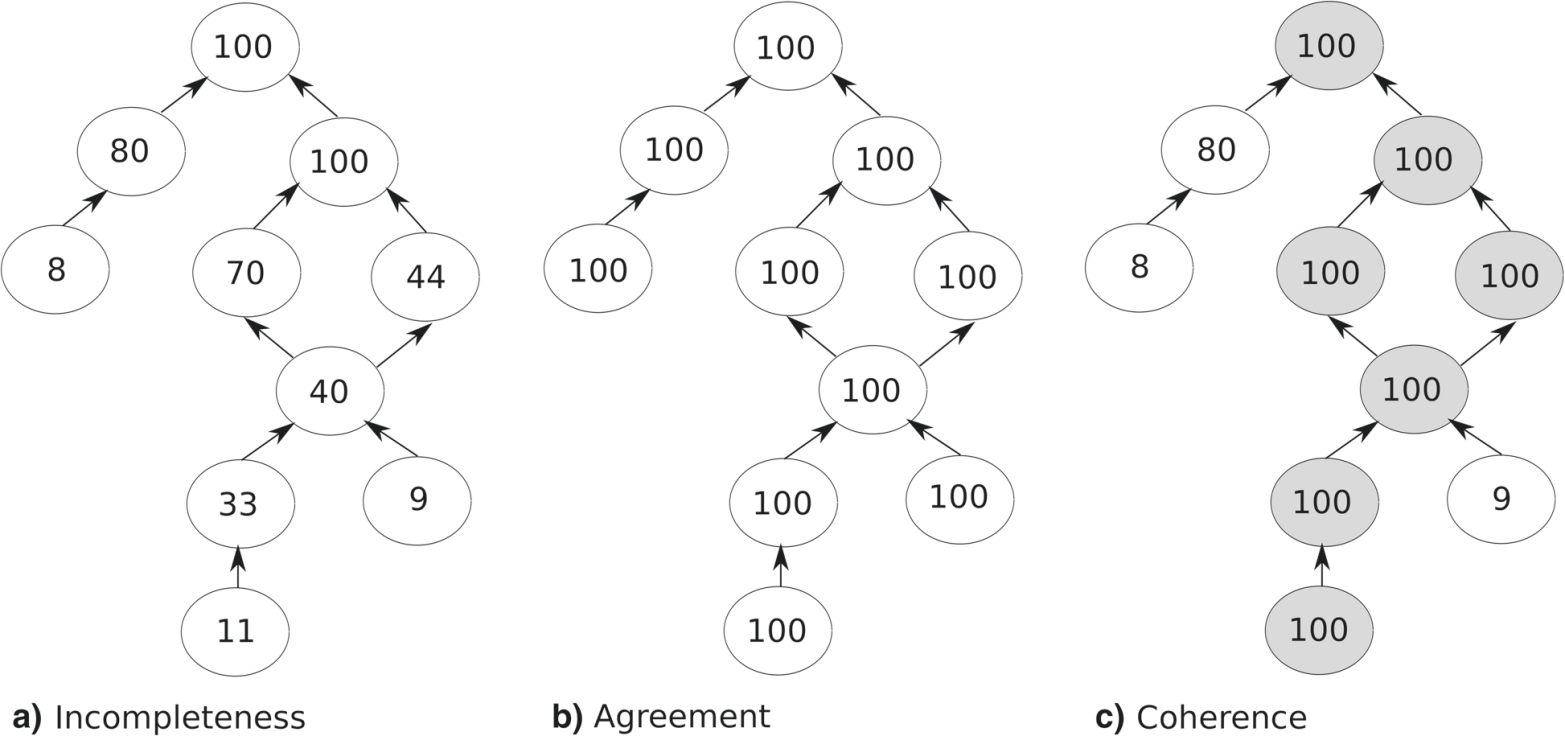
Hypothetical GO graph. Terms are represented by nodes where the number within is the number of proteins (of a given set of 100) annotated to that term. There are three situations represented: **a** annotation incompleteness, **b** annotation agreement and **c** annotation coherence

Any set of functionally related proteins, in which not all proteins are annotated to the same specificity level, can be considered to incur in a form of *annotation incompleteness*. Figure 1a) illustrates such a situation. For a hypothetical set of one hundred proteins, only one of the hypothetical annotation terms (besides the root) annotates all the proteins in that set. As the nodes get further away from the root term, it can be seen that the number of annotations dwindles until it reaches the leaf terms. And while any given protein does not need to have its most specific function represented by a leaf term, it is unlikely that a very generic term (such as a direct child of the root term) is a full descriptor of its activity. However, it is not trivial to determine this kind of incompleteness, and only after determining or predicting new functional activities can we definitively say that any given protein (or set) was incompletely annotated. Thus, in this work we limit the definition of *completeness* to the minimal set of annotations evenly distributed among the proteins in a set that characterizes the unifying functions of that set.

This kind of annotation incompleteness can derive from the fact that different protein annotation methods are used, which provide different degrees of annotation confidence. Therefore, annotation heterogeneity is created accordingly to the annotation confidence level given by each annotation method. For instance, a majority of the automatic annotation methods typically create more generic annotations. On the other hand, manual curation is more likely to lead to more highly specific annotations. Additionally, the inherent research bias towards more intensively studied model organisms and biological processes can also help further this state of incompleteness.

### Agreement

Annotation agreement can be defined as the fraction of annotations that are shared in a set of proteins. Hence, the annotation agreement of a given protein set (S) can be computed using Eq. 1 as shown below:

1 $$ annotation\ agreement\ (S) = \left(\sum_{i=1}^{t}x_{i}\right) \times \dfrac {t} {N}   $$

with *x*_*i*_ as the number of annotations for a term *i*, *N* as the total number of proteins annotated and *t* the total number of distinct annotation terms. Therefore, the greater the amount of shared annotations the greater is the annotation agreement. Figure 1b) illustrates a hypothetical full annotation agreement situation. In this situation, each one of the one hundred proteins is annotated to the same exact annotation term set and thus that hypothetical set achieves maximum or total annotation agreement. However, this is a naive metric that is also overly sensitive to annotation incompleteness and even small amounts of noise.

### Coherence

Naturally, a set of proteins having a total annotation agreement is also functionally similar, to the extent of its most specific annotation terms. On the other hand, functional similarity may not need to be so strictly defined. Additionally, due to the above mentioned incompleteness issue and the multi-functional nature of proteins, when measuring functional similarity through annotation, it may be useful to consider just some of the annotations as being functionally characteristic of a given protein set. Furthermore, for the purposes of this work, the concept of *annotation coherence* is further refined and defined as the fraction of shared annotations that define the core of the functional activities that is common and most relevant and thus able to characterize a given protein set, as a functional cohesive group. Figure 1c) illustrates a hypothetical full annotation coherence situation, where the grey shaded nodes represent the functionally more relevant terms, or the central functional cohesiveness of that set. However, a single metric is too reductive in assessing these (and other) different aspects of annotation that can dictate the functional coherence of the annotation space in protein sets. Therefore, in this work, we use a set of metrics and respective interpretation strategies relating to these three aspects of annotation described above in order to explore protein (enzyme) annotation spaces.

### Methodology

When it comes to capturing the relationship between functional and sequence similarity, the different semantic similarity metrics often present a similar behaviour, with the main distinction among them being their resolution. A comparison of several GO-based semantic similarity metrics [17], found the graph-based measure simGIC consistently showing a high resolution (and providing about 19-44 % increased resolution over the metric it derives from, the simUI metric). In the work presented here, both the simUI [18] and the simGIC [19] metrics are used for assessing functional coherence and establishing similarity baselines. Both metrics are pairwise, and as such calculate the similarity between protein pairs through their extended set of annotations (direct annotations and ancestral terms). Therefore, for a pair of proteins A and B with their extended GO term annotation sets being GO(A) and GO(B), respectively, simUI is computed by dividing the number of terms in the intersection of GO(A) with GO(B) by the number of terms in their union as shown by Eq. 2.

2 $$ simUI\left(A,B\right) = \dfrac {COUNT_{t\in \left\{ GO\left(A\right) \cap GO\left(B\right) \right\}}} {COUNT_{t\in \left\{ GO\left(A\right) \cup GO\left(B\right) \right\}}}   $$

Equation 3 is used to compute simGIC which hence is obtained by summing the Information Content (IC) of each term in the intersection of GO(A) with GO(B) divided by the sum of the IC of each term in their union.

3 $$ simGIC\left(A,B\right) = \dfrac {\sum_{t\in \left\{ GO\left(A\right) \cap GO\left(B\right) \right\}} IC(t)} {\sum_{t\in \left\{ GO\left(A\right) \cup GO\left(B\right) \right\}} IC(t)}   $$

As previously mentioned, in the [11] methodology, only the most common and specific function of a set is chosen as the most globally cohesive function. In this work it is also assumed that not all functional annotations in any given protein set (family) should characterize that set. On the other hand, considering the frequent multi-functional nature of proteins, in this work, a set of annotation terms are selected in each protein set or family as being its functional characteristic core. Therefore, the strategy employed in this work to isolate the functional characteristic cores in protein families was to resort to term enrichment analysis. In particular, a Python implementation of the ubiquitous term-for-term enrichment approach was developed. Since most of the study sets used here are relatively small, and with several terms having low expected frequencies (up to five expected observations) the Fisher exact test was used to determine enrichment. Hence, for each annotation term *t* in a given protein set a 2x2 contingency table was generated according to Table 1 with *N* being the number of proteins in the set, *M* the number of proteins in all considered sets, *nt* the number of proteins annotated with term *t* in the set and *mt* the number of proteins annotated with term *t* in all considered sets (mt). The statistical evidence of enrichment was then postulated on the basis of the *p*-values obtained from the Fisher exact test being smaller than the chosen statistical significance (alpha). It should be noted that on the term-for-term approach the graph nature of GO will lead to a statistical dependency issue. That is, for a given term annotating a certain number of proteins, at least that same number of proteins or more will also be annotated by the parental terms. Among the several strategies available to mitigate this issue, here, the Topology-based Elimination (Elim) strategy [20] was used. The strategy consists in targeting significant leaves in an annotation graph and iteratively subtracting the proteins annotated there from parent terms up to the root term. After all terms are processed new *p*-values are computed for each term. Thus, this mitigates the statistical dependencies between nodes by downplaying the statistical significance (and thus importance) of ancestor nodes. This is a desired effect, since (for a similar level of annotation quality) a more specific annotation is preferable to a general annotation. Therefore, the Elim method favours leaf terms found to be significant and at the same time removes proteins annotated to significant child terms from the parent terms annotation counts, which in turn attenuates the childrens’ influence on the parental terms. Additionally, it should be noted that the computed *p*-values for the GO terms under this strategy are conditioned on their children terms, and thus not independent. Therefore, direct application of the multiple testing theory is not possible. It is then preferable to interpret the returned *p*-values as corrected or not affected by multiple testing [20].

**Table 1:** Fisher exact test’s 2x2 contingency table

	Set	Background
annotated	nt	mt-nt
not Annotated	N-nt	(M-N)-(mt-nt)

### Coherence resilience assays

In order to test our approach Polysaccaride Lyase (PL) families of the CAZy [21] database were used as a study case. The protein (module) families in this database are organized into 5 different classes: Glycoside Hydrolases (GH), GlycosylTransferases (GT), Carbohydrate Esterases (CE), Polysaccharide Lyases (PL) and Carbohydrate Binding-Modules (CBM). The CAZy database version (c7-2011) that we used in our analysis has about 138,000 distinct UniProt identifiers distributed through the families in these classes as shown in Table 2. The performed assessments were limited to using the UniProt identifiers in those families because of their direct mapping to GO term annotations. Thus, for the annotation mapping we have used the GOA [22] annotation files (version 2013-03). Within the CAZy database the PL class is the one that is better characterized by the Glycobiology community, in part due to its more tractable dimensions, as can be seen in Table 3. For this reason we also have elected to perform our assays using this class of families. We have run the coherence resilience assays that we describe below only for families PL1 to P12, PL16, PL17 and PL22 because these are the only ones that met the minimal size requirement for our assaying.

**Table 2:** Number of protein UniProt identifiers (size) in each of the classes in the CAZy database (ver. c7-2011)

	Size
GH	70227
GT	55461
CBM	10907
CE	8110
PL	1766

**Table 3:** List of the protein families belonging to the PL class in the CAZy database and their respective size (in number of UniProt identifiers)

Family	Size	Family	Size
PL1	491	PL12	80
PL2	34	PL13	7
PL3	229	PL14	38
PL4	45	PL15	10
PL5	37	PL16	22
PL6	24	PL17	33
PL7	82	PL18	5
PL8	184	PL20	6
PL9	148	PL21	9
PL10	84	PL22	42
PL11	84	unassigned	80

**Fig. 2:**
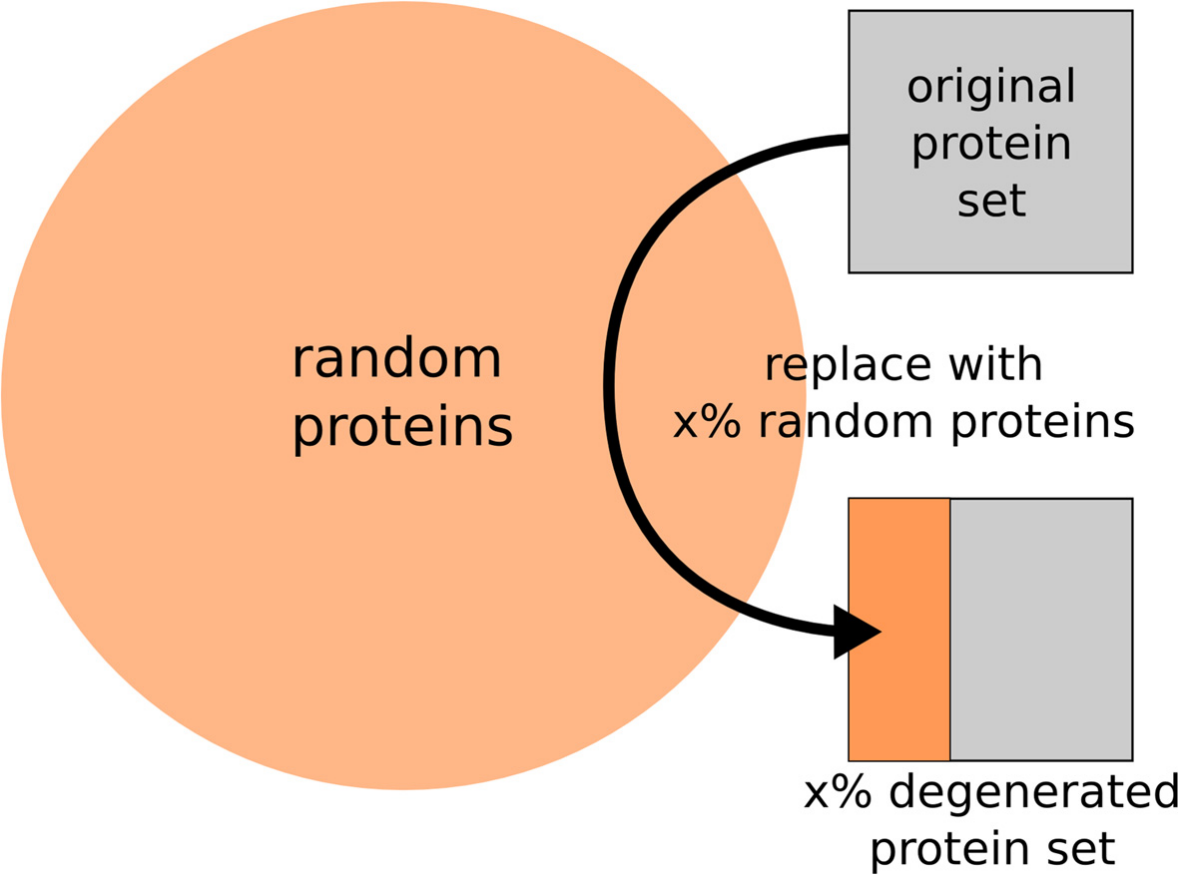
Protein set degeneration procedure. For each set (family) a chosen percentage of the set original proteins is replaced with proteins drawn randomly from outside the set

**Fig. 3:**
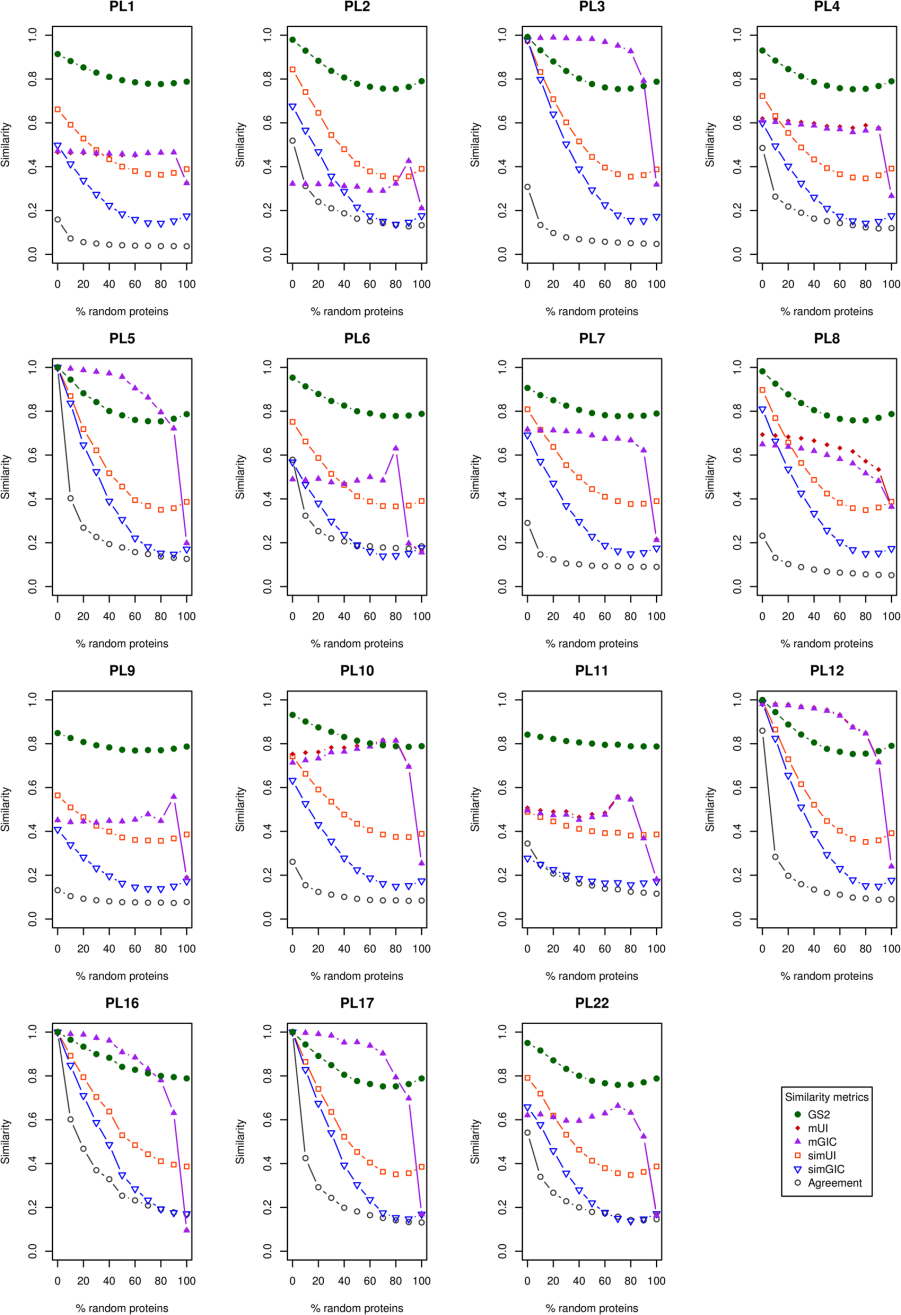
Plots of the average similarity as measured by six different metrics. For the first eight PL protein families (from the CAZy database) and their derived sets. These sets were made by replacing the original proteins with increasing amounts (of 10 % increments; 100 iterations) of random proteins (taken from the CAZy database)

In order to perform our assays we subjected each of these families (sets) to a degeneration procedure as illustrated by Fig. 2 where x % proteins in an original protein set are replaced by random proteins. In our assay these protein replacements were obtained randomly from the remainder of the CAZy families. The degeneration procedure was applied in discrete levels of 10 % protein replacement (from 0 % up to 100 % protein replacement) to each of the sets. Hence, each original protein family (0 % replacement) would gradually turn into a complete random set (100 % replacement). Consequently, for each family the functional similarity is expected to degrade progressively as the percentage of random replacement (noise) rises. Subsequently, we have used these gradual degeneration sets to assay the behaviour of each of the Agreement, simUI [18], simGIC [4] and GS^2^ [7] metrics along with two novel hybrid metrics, mUI and mGIC that we introduce further ahead. For each of the discrete levels of degeneration (noise) one hundred iterations were run per family. During each iteration, both the original family and the noise source were randomly sampled for the proteins to keep and the replacement proteins, respectively. The similarity was computed at the end of each iteration for each of the assayed metrics and then averaged for the total one hundred iterations. For all the assayed metrics (simUI, simGIC, mUI and mGIC), the global set results were obtained by averaging all the term pairwise results within each protein set. The resulting average scores are shown in Fig. 3 as plots of similarity (functional coherence) as a function of the percentage of randomly replaced proteins.

### Hybrid metrics

For this work two novel functional coherence metrics, mUI and mGIC were developed. They are based on the combination of semantic similarity metrics simUI and simGIC and a term-for-term enrichment analysis as described by the following algorithm:

1. INIT annotationGraph and annotationGraph’
2. FOR each term IN annotationGraph
3. EXECUTE enrichment analysis of term
4. IF term enriched
5. annotationGraph’ < - term
6. ENDFOR
7. mUI < - compute simUI of annotationGraph’
8. mGIC < - compute simGIC of annotationGraph’

The annotation graph for a protein set (family) being measured is generated (line 1). For each term in the annotation graph (line 2) enrichment analysis using a term-for-term (with Elim adjustment) strategy is performed as previously described. If a term is found to be statistically enriched (line 4) it is added to a derived annotation graph (line 5). When both annotation graphs are processed (line 6) the simUI and simGIC are applied to the shadow graph (annotationGraph’) resulting in the values for the mUI and mGIC metrics, respectively (line 7 and 8).

## Results and discussion

From the analysis of Fig. 3 it can be seen, as expected, that the similarity reported by each metric generally decreases as noise (in the form of random proteins) is increasingly added (replacing the original proteins) in each of the tested PL families. In this study, each of considered metrics is scaled on a [0, 1] theoretical range. The aim of our protein family degeneration assays is to observe two main aspects for each of the metrics, noise resilience and resolution. With noise resilience we check by how much the reported values can vary given the same amount of noise. As for resolution we register the difference between the maximum and minimum values it can actually report during our assays.

The Agreement metric is the least noise resilient metric, as can be seen by both the generally low values it reports and the steep declines after adding small amounts of noise to family sets with previously high agreement. This property is most evident in mono-functional families like PL5, PL16 and PL17 and also PL12 where the introduction of 10 % random proteins produces a sharp decline in the reported values. This occurs because this naive metric only equates the average of annotation term frequencies in each protein family (or set). This metric was chosen and used as the overall baseline.

The simUI and its derivative simGIC, as expected, have a similar behaviour because simGIC is a IC-weighted version of simUI. Furthermore, in the obtained results (Additional file 1) it is noticeable that simGIC presents a greater resolution than simUI (average range of 0.57 against a range of 0.46, respectively, as can be computed from Table 4), a behaviour that was also previously reported by [17] in their assessment of semantic similarity metrics. In contrast, the GS^2^ metric has the smallest resolution (for the tested sets) of all the tested metrics showing an average range of 0.18. In addition, to offering a smaller range of values (and a thus lower resolution) it is important to notice that reported values for this metric fall within the 0.75-1.0 range of similarity. Given that it is expected for protein (enzyme) families to have functionally similar proteins it would also be expected (and optimal) that these families would display higher coherence. However, when the unadulterated families are considered some of them do not provide the necessary annotations supporting such high global set functional coherence values, especially when considering values produced from the 100 % randomized sets.

**Table 4:** Difference between maximum and minimum values reported for each tested metric (Agreement, simUI, simGIC, mUI, mGIC, GS^2^) against each PL family and iterations of derived respective sets created by insertion of increasing amounts of random proteins (from CAZy) into the original families

Metrics	PL1	PL2	PL3	PL4	PL5
Agreement	0.122	0.391	0.260	0.368	0.874
simUI	0.298	0.497	0.620	0.376	0.650
simGIC	0.356	0.539	0.825	0.458	0.853
mUI	0.139	0.214	0.671	0.353	0.801
mGIC	0.147	0.216	0.672	0.343	0.802
GS^2^	0.137	0.224	0.238	0.177	0.246
Metrics	PL6	PL7	PL8	PL9	PL10
Agreement	0.405	0.201	0.180	0.058	0.178
simUI	0.386	0.432	0.548	0.207	0.368
simGIC	0.429	0.542	0.660	0.27 0	0.484
mUI	0.469	0.501	0.329	0.368	0.564
mGIC	0.474	0.505	0.285	0.372	0.559
GS^2^	0.175	0.129	0.224	0.080	0.146
Metrics	PL11	PL12	PL16	PL17	PL22
Agreement	0.229	0.771	0.831	0.869	0.400
simUI	0.108	0.644	0.613	0.649	0.443
simGIC	0.122	0.838	0.829	0.853	0.521
mUI	0.378	0.744	0.903	0.831	0.494
mGIC	0.373	0.741	0.905	0.831	0.501
GS^2^	0.054	0.247	0.211	0.248	0.191

The mUI and mGIC (such as the metrics they are derived from) also display, as expected, similar behaviours to each other. Their results measure the enrichment contribution relative to the original semantic similarity metrics. In fact, for most of the tested PL families and their respective degenerate sets the reported values are very similar. However, unlike the other tested metrics mUI and mGIC are resilient to noise (replacement with random proteins). That is evident from the gradual curves in Fig. 3 which in most families plateau until higher levels of randomization and typically only fall abruptly after addition of 90 % random proteins. This resilience to noise is conferred by the term enrichment step which pre-selects only the subset of proteins that are annotated with the terms found to be statistically significant by the enrichment procedure. Thus, this is an important factor to consider when analysing the results provided by these two metrics. As they were engineered to capture local (subset) functional coherence, for a comprehensive evaluation they should only be used in an analysis that also simultaneously considers the annotation coverage within the analysed set. This also explains the observed peaks at high noise levels in some of the families (PL2, PL6, PL9, PL11) where a small number of terms annotates a small subset of proteins and thus creates a local similarity effect. That is, at high levels of random protein replacement the original families are greatly degenerated because they lose the proteins that were characteristic for the identity of that family while, on the other hand, randomly gaining less related proteins. Hence, if a couple of random proteins being introduced happen to be very similar in terms of annotations and those terms are also found to be statistically enriched, then a new similarity core is introduced which results in the appearance of those peaks of high similarity. However, for this work this behaviour is advantageous because the underlying assumption is that each protein family shares core annotations that define the group role of that set of proteins. Thus, by using a term enrichment technique the purpose is to target and select these core annotation terms. The proteins annotated by these identified core annotation terms can then, for instance, be used for annotation extension within that set as previously proposed [23]. Thus, according to that proposal, for an hypothetical partially annotated protein set (with an expected degree of functional relatedness) the mUI/mGIC metrics can be used to identify the functional core of that set while reporting its functional similarity. If that core, reports a high similarity value and also provides enough statistical power (number of associated protein sequences) it can be used to create, for instance, a Hidden Markov Model profile model. Subsequently, that model can potentially be used as a classifier in order to extend annotations from the core to the sub-annotated sequences in the original measured protein set.

**Fig. 4:**
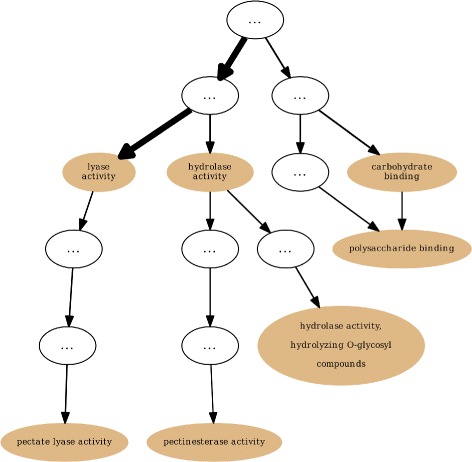
GRYFUN annotation graph. Annotation of GO molecular function ontology graph generated by the GRYFUN web application for a set of proteins from the PL10 family

Defining a completeness state and quantitatively measuring it is a challenging task considering the complexity in generalizing rules needed to detect it. Instead we approach it only qualitatively by analysing each different protein set, case-by-case by relying on domain knowledge (confirmed and expected functional associations) and then making empirical assertions about the state of annotation completeness of each protein set. For that purpose we use GRYFUN [24], a web application that we have previously developed. This application allows for annotation visualization coupled with statistical assessment (term-by-term enrichment) and is used to produce annotation graphs like the one shown in Fig. 4. The graph portrayed in Fig. 4 subsumes all the GO terms (from the *molecular function* ontology branch) annotating a set of PL10 family proteins. Unlike the typical GO graph where the edges point towards their parent terms, here the edges point towards their children and have widths proportional to the number of proteins annotated to each successive child term. The purpose is to convey the “annotation flow” and easily be able to identify “annotation bottlenecks”, or terms where annotation might have stopped despite the expectation that more proteins in that set could have been annotated to children terms of these “bottlenecks”.

For the case of the PL10 family set portrayed in Fig. 4 the “bottleneck annotation” is on the term “lyase activity”. Domain knowledge indicates that this term should annotate each protein in this family (e.g. the PL10 family is part of the Polysaccharide Lyases). However, this annotation term is relatively generic and considering the proportion of proteins not annotated with children of this term (as can be easily seen from the graph) it is fair to assume substantial annotation incompleteness. Additionally, considering the plot in Fig. 3 that represents the degeneration of the PL10 set, it can be seen that the values for mUI and mGIC actually increase along with the degeneration of the set. As previously explained the enrichment process of the mUI/mGIC algorithm considers only a protein subset of the target set being measured. Hence, it is important to consider other metrics (for instance the parent metrics simUI/simGIC) in tandem with these novel metrics for a global assessment of functional coherence in a set. Nevertheless, these novel metrics allow the identification of core activities which can potentially be extended to more sequences within the original set.

## Conclusions

Measuring the functional similarity between proteins based on their annotations is a non trivial task. Several metrics exist but due to characteristics both intrinsic to the nature of graphs and extrinsic natures related to the process of annotation each measure can only capture certain functional annotation aspects of proteins. Hence, when trying to measure the functional coherence of a set of proteins a single metric is too reductive. Therefore, it is valuable to be aware of how each employed similarity metric works and what similarity aspects it can best capture. Here we test the behaviour and resilience of some similarity metrics.

Additionally, we propose a comprehensive approach at determining functional coherence in protein sets (families) based not only on metrics but also statistics (term enrichment) and visualization coupled with domain knowledge-based empirical assessments.

Furthermore, we propose two novel metrics mUI and mGIC that combine two of the above mentioned approaches, semantic similarity metrics and term enrichment. The goal is to capture protein subsets within families (or other functionally related sets) that characterize that family (or set), which can subsequently be used for annotation extension for potentially sub-annotated proteins within the same family (or set).

The proposed approach is modular and can be integrated with other annotation methodologies mostly as a pre-processing step. In the future, we will be implementing both mUI and mGIC (along with other) metrics into our web application GRYFUN. This will more easily capture the annotation functional cores in protein sets and pipeline them to a custom annotation extension module based on HMM profiles that we are currently developing.

## Additional file

Additional file 1 Average similarity as measured by six different metrics for each of the discrete levels of noise. (XLS 50 kb)
